# Anti-TNF, a magic bullet in cancer immunotherapy?

**DOI:** 10.1186/s40425-019-0802-y

**Published:** 2019-11-14

**Authors:** Anne Montfort, Carine Dufau, Céline Colacios, Nathalie Andrieu-Abadie, Thierry Levade, Thomas Filleron, Jean-Pierre Delord, Maha Ayyoub, Nicolas Meyer, Bruno Ségui

**Affiliations:** 1 INSERM UMR 1037, Cancer Research Center of Toulouse (CRCT), 2 avenue Hubert Curien, CS 53717, 31037 Toulouse Cedex 1, France; 2 Université Toulouse III - Paul Sabatier, 31062 Toulouse, France; 3 Laboratoire de Biochimie, Institut Fédératif de Biologie, CHU Purpan, 31059 Toulouse, France; 4 Biostatistics Unit, Institut Claudius Regaud, Institut Universitaire du Cancer de Toulouse, 31059 Toulouse, France; 5 Institut Claudius Regaud, Institut Universitaire du Cancer de Toulouse, 31059 Toulouse, France; 6 Centre Hospitalier Universitaire, Institut Universitaire du Cancer de Toulouse, 31059 Toulouse, France

**Keywords:** Tumor necrosis factor, Melanoma, Anti-PD-1, Anti-CTLA-4, Infliximab, Certolizumab, Resistance, Immune-related adverse events

## Abstract

Immune checkpoint blockers (ICB) have revolutionized cancer therapy. However, complete response is observed in a minority of patients and most patients develop immune-related adverse events (irAEs). These include colitis, which can be treated with anti-tumor necrosis factor (TNF) antibodies such as Infliximab. In a recent issue of the Journal for ImmunoTherapy of Cancer, Badran et al. reported that co-administering Infliximab together with ICB to five cancer patients prevents colitis recurrence, with four of them exhibiting overall disease stability. The basis for this treatment strategy stemmed from our pre-clinical demonstration that TNF contributes to resistance to anti-PD-1 therapy. In agreement with this concept, we have shown that TNF blockers improve the anti-tumor therapeutic activity of ICB in mice and based on these findings we are currently evaluating the combination in melanoma patients enrolled in the TICIMEL clinical trial. Herein, (i) we discuss the scientific rationale for combining anti-TNF and ICB in cancer patients, (ii) comment on the paper published by Badran et al. and (iii) provide the TICIMEL clinical trial design.

Melanoma patients can currently be considered as the ones who benefited the most from ICB therapy, although about 60% of patients relapse within three years following treatment induction [1]. While boosting anti-tumor immune responses, these therapies are also responsible for the occurrence of immune-related adverse events (irAEs) with some of them, such as colitis, being treated with TNF-blocking antibodies. In particular, Infliximab, a first-generation chimeric TNF blocking monoclonal antibody, can be used in the clinic to treat ICB-induced colitis in cancer patients who do not respond to corticotherapy. The standard protocol is to administer one (or two) bolus of Infliximab after ICB therapy discontinuation [2]. Approximately, 1% of patients with advanced melanoma treated with ICB develop severe colitis, which requires Infliximab treatment. Interestingly, one Infliximab infusion can efficiently cure colitis in most patients, without impacting melanoma outcome [2]. In a recent article, Badran et al. described a small retrospective series of 5 patients affected with various cancers and treated with ICB (including 2 patients with Ipilimumab and Nivolumab combination) [3]. All patients had developed severe corticosteroid-resistant colitis justifying the introduction of Infliximab therapy. In contrast with the standard protocol of colitis management, the authors continued the ICB therapy while co-administering Infliximab. Whereas all patients displayed reduced colitis symptoms, overall disease stability was observed for all but one of the five patients [3].

The authors notably based their rationale for such a combination on observations we made, supporting the use of TNF blocking agents to promote the efficacy of ICB in cancer and especially melanoma. In a mouse melanoma model, we demonstrated that TNF impairs the accumulation of CD8^+^ T cells in tumor-draining lymph nodes and tumors in a TNFR1-dependent manner. This was associated with the ability of TNF to induce activation-induced cell death (AICD) of CD8^+^ T cells thus promoting tumor growth and impeding response to anti-PD-1 [4–6]. These results led us to demonstrate the benefit of using TNF-blocking antibodies to potentiate the therapeutic effects of anti-PD-1 in melanoma-bearing mice going from 20% tumor rejection with anti-PD-1 alone to 75% with the combination therapy [6, 7]. Mechanistically, TNF blockade prevented anti-PD-1-induced AICD of tumor-infiltrating lymphocytes (TILs) and decreased their PD-L1 and TIM-3 expression. Recently, Perez-Ruiz E. and co-workers extended the concept by showing the role played by TNF in promoting AICD of CD8^+^ TILs upon anti-PD-1 and anti-CTLA-4 combination therapy in mice [8]. They also illustrated the therapeutic efficacy of the combination in other mouse cancer models (MC38 and HT29 colon cancer and B16-OVA melanoma models) and demonstrated the efficient control of inflammatory bowel disease (IBD) symptoms by TNF blocking agents in mice [8].

In their work, Badran et al. concluded that combining immunotherapy to Infliximab in order to treat cancer patients while managing irAEs is safe and does not negatively impact anti-tumor efficacy [3]. Whereas we found this article of interest for the cancer and immunotherapy fields, several methodological weaknesses limit the interpretation of such results. First, the small number of patients and the variability of tumor histological types as well as that of ICB regimens, some of which including targeted therapy, chemotherapy or radiotherapy with all of them being administered in the absence of standardized therapeutic protocols, do not allow for definitive conclusions as regard to the safety of any combination. Moreover, several studies have reported that patients developing irAEs, including colitis, may be more inclined to display objective response to ICB. Since all patients included in this cohort received anti-TNF following the emergence of irAEs, the impact Infliximab has on ICB response in cancer patients cannot be extrapolated. This can be related to the fact that the study is based on a retrospective analysis, which may have led to biases in building the cohort analysis. Finally, the authors explain that the choice to maintain anti-TNF treatment was motivated by the desire to rapidly reduce corticosteroid therapy and to maintain treatment with ICB. However, clinicians experienced in the use of ICB have noticed the often rapid and durable efficacy of anti-TNF agents in the treatment of colitis, sometimes with a single injection. It has also been reported that patients may be re-exposed to ICB after a medicated irAE has been resolved, without systematic recurrence of side effects. The above-mentioned concerns reduce the scope of the clinical observations reported in this article, and question the relevance of exposing patients, outside of clinical trials, to a therapeutic regimen extrapolated only from data based on mouse models. Notwithstanding these considerations, the study published by Badran et al. indicates for the first time that concurrent treatment of cancer patients with ICB and anti-TNF not only prevents ICB-induced colitis but also facilitates steroid tapering. Considering that steroids likely impair anti-cancer immune responses, anti-TNF may constitute a good alternative strategy to prevent a subset of irAEs triggered by ICB. Whereas the work of Badran et al. seems to confirm the efficacy of Infliximab administration to treat colitis in cancer patients under ICB therapy in agreement with a recent retrospective clinical study [2], the consequences of anti-TNF and ICB combination on other irAEs and putative suspected unexpected serious adverse reactions (SUSAR) warrants further investigation. Furthermore, the impact Infliximab has on the anti-cancer immune response has not been addressed in the study by Badran and colleagues. Considering the dual role TNF plays in anti-cancer immune responses, this point remains a critical issue that needs to be carefully evaluated.

Building on our preclinical findings [4–7], we initiated at Toulouse Oncopole a phase 1b clinical trial (TICIMEL -NCT03293784) in 30 advanced melanoma patients to investigate the concomitant administration of Ipilimumab (anti-CTLA-4), Nivolumab (anti-PD-1) and anti-TNF (Infliximab or Certolizumab) (Fig. 1). The primary objective is to evaluate the safety and tolerability of this combination. Among TNF blocking agents already available in the clinic, we selected Infliximab and Certolizumab for combination with ICB (Fig. 1). As indicated above, Infliximab is already used for treating ICB-induced colitis. As a full IgG1 chimeric antibody, Infliximab may induce an Fc fragment-dependent depletion of membrane TNF-expressing leukocyte populations [9]. Certolizumab is a pegylated Fab’ fragment targeting TNF, devoid of an Fc fragment and shown to be protective in rheumatoid arthritis and Crohn’s disease patients [10]. We did not select Etanercept as this molecule can also bind lymphotoxin alpha. This trial is designed to assess for the feasibility of the combination therapy in patients, and will provide significant clues of its efficacy. Moreover, a dedicated ancillary part aims at evaluating the immune response in blood and tumor bed along therapy.

**Fig. 1:**
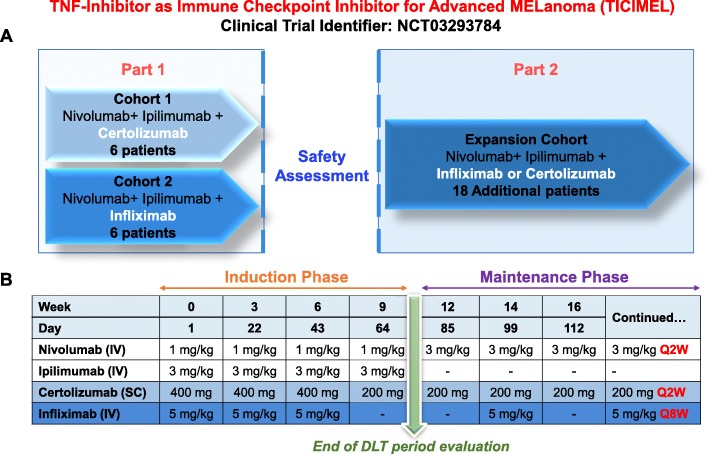
Scheme of the TICIMEL phase-1b clinical trial in 30 advanced melanoma patients. **a**, TICIMEL is split in 2 consecutive parts with the first part being conducted in 2 parallel cohorts (Cohort 1 and Cohort 2 with alternative patient allocation) to evaluate the safety profile of combining Nivolumab+Ipilimumab with TNF-Inhibitors (Certolizumab in cohort 1 and Infliximab in Cohort 2). Three patients are included at the unique dose. If there is no DLT or only one DLT, three other patients will be included. If no more than one patient among 6 presents a DLT, the combination (ICB + anti-TNF) will be considered as safe and allow to pursue the second part of the trial. The combination therapy selected for the second part of the study (cohort expansion study) will depend on safety, activity, and pharmacodynamics data from the first part of TICIMEL. **b**, Nivolumab and Ipilimumab are administered intravenously (IV) (infusion duration of 60 min for Nivolumab and 90 min for Ipilimumab); Certolizumab is administered subcutaneously (SC). Infliximab is administered IV (infusion duration of 120 min). All treatments are given on the same day as indicated in the induction phase. During the maintenance phase, Nivolumab and Certolizumab or Infliximab are/will be co-administered as indicated. Patients undergoing disease control (CR, PR or stable disease) beyond one-year treatment will have the possibility to be maintained on Nivolumab (3 mg/kg, Q2W). The end of Dose Limiting Toxicity (DLT) period evaluation is at day 84

To conclude, the article from Badran et al. furthered findings regarding the management of ICB-induced colitis by TNF blockade [2, 3]. Nonetheless, TICIMEL will allow for the assessment of the safety of combining anti-TNF and ICB in cancer patients within a clinical trial specifically designed for this purpose. In addition, it will set the grounds for putting forward this combination in future more advanced-phase trials seeking proofs of efficacy.

## Data Availability

Not applicable.

## Abbreviations

AICD: Activation-induced cell death
CTLA-4: Cytotoxic T-lymphocyte-associated protein 4
DLT: Dose Limiting Toxicity
IBD: Inflammatory bowel disease
ICB: Immune checkpoint blockers
irAEs: immune-related adverse events
IUCT-O: Institut Universitaire du Cancer de Toulouse-Oncopole
PD-1: Programmed cell death 1
PD-L1: Programmed cell death ligand 1
SUSAR: Suspected Unexpected Serious Adverse Reactions
TILs: Tumor-infiltrating lymphocytes
TIM-3: T-cell immunoglobulin and mucin-domain containing-3
TNF: Tumor Necrosis Factor alpha
TNFR1: Tumor Necrosis Factor Receptor 1
